# Supplementary material for: Are carbon emissions trading and green financial instruments synergistic? -Comprehensive quantitative research based on content analysis
Source: PLoS One. 2024 Mar 7;19(3):e0298601. doi: 10.1371/journal.pone.0298601 (PMC10919601; doi:10.1371/journal.pone.0298601)
Supplement: S1 Data — (ZIP) [file pone.0298601.s001.zip › Data/地级市Z_F工作报告.zip!/┴╔─■/┤≤┴1⁄4╩╨/└┤╫╘╣1⁄2╓┌║┼í╛┬φ┐╦╩2╛▌═°í┐_2010.docx]

各位代表：

　　现在，我代表市政府向大会报告工作，请予审议，并请市政协各位委员提出意见。

　　一、2009年政府工作回顾

　　2009年，是新世纪以来我市经济发展最为困难的一年。在党中央、国务院和省委、省政府及市委的正确领导下，市政府团结带领全市人民，坚定信心，迎难而上，努力克服国际金融危机影响，“保增长、保民生、保稳定”取得重大成绩，圆满完成十四届人大二次会议确定的各项任务，实现了主要经济增长指标“两个高于”的目标。

　　──经济保持平稳较快增长。预计全市实现地区生产总值4410亿元，增长15%；完成地方财政一般预算收入400.2亿元，增长18%；完成规模以上工业增加值1733亿元，增长17.5%。

　　──对外开放再创新优势。进出口贸易总额403亿美元；实际使用外资增长20.2%，总额达60.2亿美元，再列全省第一。

　　──可持续发展能力不断增强。万元GDP综合能耗下降5.7%，主要污染物排放总量平均削减5%以上。完成全社会固定资产投资3273亿元，增长30%。

　　──民生质量进一步提高。实现城镇就业16万人，登记失业率为2.8%。城镇居民人均可支配收入19070元，增长9%；农民人均纯收入11190元，增长14%。

　　一年来，市政府主要做了以下几方面工作：

　　（一）全力以赴打赢保增长攻坚战

　　狠抓投资拉动。全年共汇集投资1000万元以上建设项目1400多个，总投资规模达1.28万亿元。实施10大基础设施建设工程、10大民生工程、10大新农村建设工程和一批重大产业项目。地铁、英特尔芯片工厂等75个重点项目顺利推进，新机场、跨海通道等20个项目的前期工作全面展开。获得中央新增投资项目225个、补贴资金9.6亿元。

　　积极扩大消费需求。实施促进房地产市场健康稳定发展、商业促消费、旅游业提振市场等政策措施，开展“家电、汽车下乡”和繁荣城乡市场系列活动，全市商品房销售额增长51.8%，社会消费品零售总额增长18.1%，旅游总收入增长14.8%。

　　千方百计稳定出口。实施出口多元化战略，稳定传统市场，开拓新兴市场，船舶出口逆势上扬，软件出口及服务外包加速增长。加大政策扶持力度，扩大中小企业国际市场开拓资金适用范围，提高出口信用保费补贴。全年实现外贸出口208亿美元，同比下降12.7%，降幅低于全国和全省平均水平。

　　保障要素供应。组织新增信贷投放1442亿元，利用资本市场融资37.7亿元，加强煤、电、油、运的调度和管理，妥善解决发展用地制约。加强区域电网建设改造，投资34.3亿元，增长36.1%；投产线路798公里，增长80%；容量增长96%，新增容量相当于1992年全市总规模。

　　全力保持企业平稳较快发展。实施支持中小企业稳定健康发展优惠政策和措施，组织开展“机关干部走进千家重点企业”活动和中小企业融资促进行动，全年为企业补贴、减负30多亿元，落实支持资金453亿元，新增中小企业贷款537亿元。全市企业付出了比以往更大的艰辛和努力，在保增长、保就业中发挥了重要作用。

　　加快发展县域经济。坚持“多予、不取、放活”方针，全市财政用于“三农” 支出44.7亿元，增长24.9%。县域重点工业园区完成投资468.3亿元，实现工业增加值130.3亿元。积极发展现代农业，新建设施农业11万亩、省级渔业健康养殖示范区项目45个、规模化畜牧饲养小区122处、标准化示范果园55万亩。农业全面丰收，粮食产量达30.7亿斤，肉蛋奶渔果产量再创新高。新农村建设15件实事、90项任务全部完成。涉农区（市）县主要经济指标增幅高于全市平均水平。

　　（二）深入推进改革开放

　　制定落实国家战略的规划措施。《辽宁沿海经济带发展规划》上升为国家战略对大连振兴发展具有里程碑意义。我们抢抓机遇，统筹谋划，制定了贯彻落实的实施意见和专项规划，为加快区域核心城市建设描绘了宏伟蓝图。

　　加快航运中心建设。鲇鱼湾港区22号原油泊位、长兴岛疏港路主体工程、大窑湾疏港路港区段等工程竣工，机场三期扩建工程顺利推进，长兴岛深水港、大连湾疏港路开工建设。大连港与锦州港的战略合作进一步加强，长春、哈尔滨等内陆干港建设全面推进。海港完成货物吞吐量2.7亿吨，集装箱吞吐量457.6万标准箱，分别增长10.6%和1.1%。空港完成旅客吞吐量955万人次，增长16.4%。

　　努力提高开放水平。先导区和重点产业园区引领作用更加突出，旅顺、长海全面对外开放，庄河港、旅顺新港通过对外开放验收，庄河临港经济区等4个区域被纳入辽宁沿海经济带重点发展和支持区域。全市利用外资规模和质量不断攀升。引进内资647.5亿元，增长57.6%。成功举办夏季达沃斯年会、“北京（大连）—东京论坛”和国际服装节。

　　稳步实施重点领域改革。市、区两级政府机构改革基本完成。大商、国合、盛道、盐业公司等国有企业股份制改革顺利推进，组建了大连装备制造投资有限公司。民营经济继续发展壮大，增加值占地区生产总值比重提高到57.9%。全市农村集体土地流转面积13.3万亩，集体林权制度主体改革任务基本完成，设立3家村镇银行和15家小额贷款公司。甘井子区综合配套改革继续深化。

　　（三）加快构建现代产业体系

　　强化优势产业集聚效应。石化、装备制造、造船、电子信息与软件四大产业支撑作用进一步增强。中远造船、固特异轮胎、一重核岛主设备等项目顺利推进，PTA、PX、甲醇项目投产，重工·起重风电扩能改造等9个项目列入国家产业振兴和技术改造计划，90型船用曲轴等一批具有领先水平的产品下线。瓦房店风电设备制造基地建成投产，保税区国际能源港初具规模，长兴岛造船基地、花园口新材料产业基地、旅顺机车产业园建设开端良好，新能源汽车项目加速推进。奇瑞、曙光整车项目落户大连，填补了我市汽车产业的空白。

　　推进高新技术产业规模化发展。实现高新技术产品增加值1052亿元，增长31.2%。旅顺南路软件产业带全线拓展，东软河口国际软件园、腾飞软件园二期投入使用。数控、光电子、新能源产业发展速度加快，我市成为全国节能与新能源汽车、半导体照明应用和“金太阳”示范试点城市。

　　加快发展现代服务业。星海湾金融商务区一期工程投入使用，渣打银行、百年人寿等23家内外资金融机构和投资公司开业，大连银行等金融总部开设9家异地机构，大连商品交易所实现交易额37.6万亿元。总部经济、现代物流、商贸流通业快速发展，东港商务区、小窑湾国际商务中心建设加快。金渤海岸、长山群岛等旅游度假区建设扎实推进。

　　实施创新驱动战略。17个项目获国家“高档数控机床与基础制造装备”重大科技专项支持。全市引进海外研发团队12个，收购国外科技型企业10家。申请发明专利2388件，增长34%。高新区成为“海外高层次人才软件创新创业基地”。

　　（四）全面提高城乡规划建设和管理水平

　　科学调整城乡建设规划。基本完成城市总体规划、土地利用规划和区域用海规划修编工作，在调整空间布局、推动集约发展方面迈出实质性步伐。

　　加强基础设施建设。城市10大交通工程进展顺利，东联路等项目竣工。哈大铁路客运专线大连段主体工程基本完工，滨海大道大连段860公里全线贯通，202路轨道延伸线开工，改建普通公路190公里，新建农村公路500公里。大伙房水库输水入连工程和三道沟净水厂改造加快实施，除险加固水库32座。甘井子热电厂、华能电厂改扩建工程开工，开发区热电厂获国家核准。国际会议中心、体育中心体育馆建设顺利。城建“五个一”工程完成124个项目。

　　注重生态环境改善。全面启动森林城市创建工作，实施滨海大道等八大绿化工程，植树8067万株。治理生态河226.6公里。植物园、白云雁水健身公园、西山揽胜景区改造和滨海路人行木栈道建设竣工。城市绿化覆盖率达到44.5%，人均公共绿地面积增至12.3平方米。启动百万平方米环境友好型住宅示范工程建设，322台节能与新能源汽车投入使用。关闭粘土砖厂58家。建成19座污水（污泥）处理厂，城市污水处理率达到90%以上。全年城市空气质量优级天数创有监测历史以来最好水平。在国际花园城市评选中，大连摘得最高级别组桂冠。

　　提高城市管理水平。开展市容环境综合整治200天行动，在全市开展城市管理考核工作，城市管理水平进一步提升。

　　（五）切实加强以民生为重点的社会建设

　　扎实推进民生工程。制定实施14项稳定和扩大就业政策措施，实施特别培训计划，就业服务平台向农村延伸，沙河口区大学生创业就业中心成为全国示范工程。建立城乡低保标准自然增长机制，企业退休人员养老金月人均达1288元。累计投入医疗救助资金3409万元，救助困难群众9.4万人次。市孤残儿童康复中心主体竣工，重度残疾人托养中心投入使用，全国首家残疾人信息化就业基地运营。慈善事业健康发展。构建多层次住房保障体系，通过货币补贴方式安排经济适用住房8000户，廉租房保障范围不断扩大、补贴标准进一步提高。稳步提高职工住房公积金积累，增强中低收入家庭改善住房能力。完成农村困难居民危房改造800户，生态移民搬迁700户。建设农村安全饮水工程75项，8.8万人用上安全自来水。新建农村户用沼气1.3万户、大中型沼气工程50个。完成农村26个新社区、15个新村庄建设试点。建设农村社区服务中心440个，促进了城乡公共服务均等化。

　　积极发展社会事业。加快完善教育设施，新建、扩建校舍25.7万平方米，偏远农村寄宿制学校建设项目顺利推进，为农村高中新建实验室53个，实施中小学免费体检计划和健康饮水工程，为8000余名家庭困难的高中生每人发放助学金1000元。我市荣获全国首批义务教育均衡发展先进地区称号。启动医药卫生体制改革，城市社区卫生服务体系进一步完善。防控甲型H1N1流感取得明显成效。农民参加新型农村合作医疗范围进一步扩大，人均筹资标准提高到140元。率先在全国实现计划生育国家级优质服务区（市）县全覆盖。全市新建乡镇综合文化站52个、农家书屋500个，农村文化信息资源共享工程入户50万个。文化艺术活动丰富多彩，电视剧《闯关东》荣获全国“五个一工程”奖。成功举办第11届市运会，大连籍运动员在第11届全运会上为辽宁代表团夺得的金牌数列全省各市首位。

　　全力维护社会稳定。圆满完成国庆60周年、夏季达沃斯年会和服装节安保任务。维护政治稳定、打击犯罪长效机制和社会治安防控体系不断完善，信访接访调处能力增强，一批影响社会稳定的案件得到有效化解。城市社区“两委”换届选举工作圆满完成，西岗区等24个单位成为国家、省和谐社区建设示范单位。“食品安全城市”建设八大工程深入实施。应急平台体系建设扎实推进，应急管理能力得到加强。深入开展安全生产执法、治理、宣教三项行动，沙河口区、中山区获“全国安全社区”称号。

　　民族、宗教、侨务、对台、人防、气象、防震减灾、档案、老龄、双拥共建、民兵和预备役等工作取得了新成绩。

　　（六）努力建设服务型政府

　　深化政府管理创新。行政审批项目进一步缩减，网上审批项目达到50%。建成市政府数据中心，政务外网正式开通运行。行政投诉办结率和群众满意率达95%以上。市区两级建立依法行政制度377项。ISO9000质量管理体系认证工作成果不断巩固。

　　加强政风行风建设。强化对节能减排落实、土地执法监督检查，深入开展工程建设领域突出问题专项治理。加大对政府投资项目和重点建设资金的跟踪审计力度，提高了财政资金的使用效益。开通24小时“行风热线”，拓宽了政府与广大市民的沟通渠道。

　　认真接受监督。自觉接受人大及其常委会的法律监督、工作监督和政协的民主监督，坚持重大事项向人大报告、向政协通报制度。定期听取人大代表、政协委员和各民主党派、工商联以及无党派人士的意见和建议。694件人大代表建议和684件政协提案全部办结，满意率达95％以上。

　　各位代表，回顾过去的一年，我们以坚定的必胜信心，勇于直面困难，高标准确定目标，强力度落实政策，快节奏推进工作，赢得了经济形势回升向好的有利局面；以昂扬的进取精神，抢抓机遇，开拓创新，不断强化核心地位和龙头作用，推动大连进入大开发、大开放和大发展的新阶段；以科学的发展理念，统筹兼顾，促进和谐，使全市经济社会可持续发展能力显著增强，人民群众的生活水平不断改善；以强烈的责任意识，勤奋敬业，求真务实，埋头苦干，在振兴发展的伟大实践中创造了可喜的业绩。

　　我们深感成绩来之不易！这些成绩无不凝结着全市人民的心血和汗水，无不体现着社会各界的帮助和支持。在此，我代表市政府，向齐心推动大连发展的全市人民，向大力支持政府工作的人大代表和政协委员，向各民主党派、工商联、人民团体和无党派人士，向离退休老同志，向中省直各部门和兄弟省市驻连机构，向驻连部队和武警官兵，向关心支持大连建设发展的海内外朋友表示衷心的感谢！

　　在充分肯定成绩的同时，我们还要清醒地看到当前存在的困难和问题：国际金融危机的影响还没有完全消除，需求不足导致工业经济增长面临诸多困难，经济结构调整与发展方式转变的任务仍很艰巨，外资外贸形势依然严峻，城市空间对发展的制约日渐凸显，就业等一些事关民生的工作压力较大，城市居民收入增长不快，政府一些部门和工作人员服务意识和水平有待进一步提高。对此，我们必须高度重视，切实加以解决。

　　二、2010年政府工作目标与任务

　　今年是“十一五”规划的最后一年，也是全面落实《辽宁沿海经济带发展规划》和应对国际金融危机的关键之年。虽然外部经济环境不确定不稳定因素仍然较多，但从总体上看，今年的经济发展环境将好于去年。特别是辽宁沿海经济带开发开放和国家进一步实施东北老工业基地振兴战略为大连振兴发展赋予了新使命、注入了新动力。我市多年积蓄的能量，为实现又好又快发展提供了强大的后劲。只要我们始终保持坚定的信念和昂扬向上的精神状态，把形势判断得更准确一些，把困难估计得更充分一些，把措施安排得更周密一些，高标准、高效率、高水平地抓好各项工作，就一定能够在新一轮增长中把握主动，就一定能够在率先全面振兴中大有作为，就一定能够在科学发展的实践中再创佳绩！

　　今年政府工作的总体思路是：认真贯彻党的十七届三中和四中全会、中央经济工作会议、省委十届九次全会和市委十届八次全会精神，坚持以科学发展观为统领，抢抓加快东北振兴和辽宁沿海经济带开发开放的双重历史机遇，推动经济结构调整和发展方式转变，全力推进全域城市化进程，加快“三个中心、一个聚集区”建设，不断提高经济增长质量和效益，全面促进民生改善和社会和谐稳定，为率先全面振兴、实现科学发展新跨越奠定更加坚实的基础。

　　经济社会发展的主要预期目标是：地区生产总值增长15%以上，突破5000亿元；万元GDP综合能耗降低4%，主要污染物减排3%；地方财政一般预算收入增长15%；全社会固定资产投资增长30%以上；实际使用外资、引进内资均增长20%以上，外贸自营出口增长10%；社会消费品零售总额增长15%以上；城镇居民人均可支配收入增长10%，农村居民人均纯收入增长12%；城镇登记失业率控制在3.5%以下。

　　今年市政府工作的主要任务是：

　　（一）着力推进全域城市化，拓宽跨越发展战略空间

　　实施全域城市化发展战略，是全市上下在振兴发展进程中，对突破资源、空间制约形成的广泛共识，是落实国家战略，拉开发展框架的必然选择，也是当前调结构、扩内需，加快发展方式转变的重要途径。我们必须立足当前，着眼长远，实施建设区域核心城市规划，研究编制“十二五”规划，加快推进全域城市化进程，实现工业化和城市化相互促进，打造经济社会又好又快发展的强力引擎。

　　以全域城市化拓展空间、优化布局。按照组团式、链条式发展的思路，沿黄渤两海、沈大和丹大两线拓展城市空间，规划建设“四大组团”，立足现有基础和优势，明确产业发展方向和城市定位，加快形成各具功能特色的经济区和生态环境优美的宜居区。主城区组团重点发展现代服务业，打造企业总部密集的国际商务区、软件和创意产业发达的知识经济聚集区和科教先进、文化繁荣、环境优美的生态宜居城区。新市区组团重点发展先进制造业、物流业、总部经济、高新技术产业，建设航运服务资源聚集区、先进制造业聚集区、临海临港装备制造业基地和宜居新市区。渤海区域城市组团重点发展石化、造船、轴承、重大装备制造、滨海旅游等产业，建设临港产业的新增长极和渤海湾畔新兴城市。黄海区域城市组团重点发展静脉、光伏、新材料、家具制造、海洋水产和旅游度假等产业，建设国家级新材料产业基地、国际旅游胜地和区域重要节点城市。

　　以全域城市化拉动投资、扩大消费。推动制造业布局向北转移，基础设施建设向北延伸，重大项目和生产要素向北聚集，使新市区和黄渤海组团成为投资的热土。提高全域投资强度，着力引进和建设一批投资额大、拉动力强、影响深远的基础设施和产业项目，全社会固定资产投资向5000亿元的目标迈进。加大资金筹措力度，积极引导金融机构扩大信贷投放。充分激发全域城市化对促进消费的拉动作用，千方百计提高居民收入水平，完善城乡市场体系，促进节庆消费，拓展旅游、会展消费，培育消费新热点。深入推进“家电、汽车、农机下乡”和“农超对接”，扩大农村消费。推进市区、新兴城市组团和小城镇三点联动，大力发展县域住宅、商业和工业地产，促进房地产业健康发展，加快城市化步伐。

　　（二）着力抓好“三个中心”建设，强化核心城市功能

　　认真组织实施东北亚国际航运中心、东北亚国际物流中心和区域性金融中心建设规划，加快完善城市功能，增强城市的辐射力和带动力。

　　全面提升航运中心功能。制定完善港口、经济区、城镇联动建设和发展规划，加快北黄海港口建设和开发开放步伐，形成黄、渤两海星罗棋布的港口群和贯穿全域的城市带、经济带。推进大窑湾三期码头、长兴岛油码头等重大港口基础设施建设，打造现代港口集群。加快长兴岛、大连湾疏港路建设，进一步推进跨海通道项目。深化港口管理体制和大连港集团改革，提高管理水平和服务效率。海港货物、集装箱吞吐量均增长15%以上。大力引进国际知名港航企业，积极开辟新的国际班轮航线。加快机场三期改造，空港旅客吞吐量突破千万人次；推进新机场前期工作。

　　不断完善物流中心架构。推进大窑湾铁路集装箱中心站和内陆干港建设，争取东北海铁联运体系纳入国家多式联运示范工程。加快保税区汽车物流城建设，申办建设长兴岛综合保税区。规划建设油品、矿石、粮食、煤炭、冷链、钢材等国际标准化物流基地，培育和引进一批具有国际竞争力的现代物流企业。

　　加快推进金融中心建设。高水平建设人民路和星海湾金融商务区。支持大商所上市期货新品种，建设亚洲期货交易中心。加快东北亚煤炭交易中心建设。健全现代金融服务体系，积极设立和引进各类金融机构。促进驻连金融机构发展壮大，支持大连银行、百年人寿等金融总部加快全国网络布局。大力发展多层次资本市场，推动企业上市和直接投资。

　　（三）着力构建现代产业聚集区，推动结构调整和发展方式转变

　　落实产业振兴规划，转变发展方式，加快构筑以绿色、低碳经济为方向的具有国际竞争力的现代产业聚集区。

　　做强做大优势产业集群。注重以高新技术改造传统产业，巩固提升装备制造、石化、船舶等产业的优势地位。以建设世界级产业基地为目标，继续推进长兴岛和旅顺船舶基地、核电装备基地、机车产业基地、奇瑞汽车等一批重大项目建设。推动资源要素向优势产业聚集，培育建设开发区石化、开发区和保税区汽车、高新区软件与服务外包、花园口新材料等10个产值500亿元以上的产业集群，松木岛化工、三十里堡船舶配套等10个产值200—500亿元的产业集群。

　　加快培育新兴产业。重点发展新能源装备、节能与新能源汽车、软件与服务外包、新材料及生物等新兴产业。加快英特尔芯片、新能源汽车等项目建设，实施大连检测科技园、高新区欧力士服务外包基地、开发区和瓦房店光电等一批新兴产业项目和示范工程，推动全市产业结构优化升级。

　　发展壮大现代服务业。大力发展物流、商贸、金融、旅游等高附加值服务业。借助夏季达沃斯年会品牌效应，发展以星海湾、钻石海湾、金石滩和长海为基地的会议经济。推动总部经济、楼宇经济、文化创意、中介服务等城区经济发展。编制黄渤海旅游经济圈发展规划，重点推进金渤海岸、长山群岛、三山岛、旅顺阳光海岸、瓦房店龙门和驼山、普兰店安波、庄河步云山等旅游度假区建设。加快小窑湾国际商务中心区、西城国际旅游商务区、体育新城等现代服务业聚集区和高新区万达广场、动漫走廊、鲍鱼湾文化影视等创意产业基地建设。

　　切实增强科技创新能力。建设国家大型轴承工程技术研究中心等一批高水平实验室、工程中心和中试基地，完善企业与在连高校、科研院所的技术创新联盟，建立国家级新能源、数控机床等创新基地，加快区校一体化进程。高标准规划建设大连生态科技创新城，推进中科院大连科技创新园和都市科技园建设。落实知识产权战略纲要，培育自主品牌。推进开放式创新，大力引进海外研发团队，并购科技型创新企业。发挥“海外学子创业周”、欧美同学会大连报国基地作用，办好“软交会”、“专交会”，集聚高端人才、技术等创新要素。

　　（四）着力深化改革开放，增强发展活力与动力

　　进一步深化改革、扩大开放，努力破解发展难题，加快形成有利于提升核心地位和龙头作用的体制机制。

　　深化经济体制改革。推进国有大型企业兼并重组和股份制改革，完善国有资产监督管理体系，扩大国有产权进场交易的覆盖范围。加快民营经济发展，落实促进中小企业发展的各项政策措施，放宽领域限制，降低准入门槛，切实加强政策指导、要素保障和协调服务。

　　抓好重点领域和关键环节改革。深化政府机构改革，完善行政管理体制。落实国家资源性产品价格和环保收费改革措施。改革公共财力配置方式，建立健全以项目决定资金投入的立项、审批、拨付机制，集中财力办大事。规范地方政府事权和财权范围，推进“乡财县管”改革。推动农村信用社改制组建市农村商业银行，新设5家中小银行和15家小额贷款公司。根据全域城市化的需要，探索建立适应功能区和行政区融合发展的区域管理体制。积极推进农村土地、林地承包经营权流转，搞好集体林权配套改革，大力发展农民专业合作社。支持甘井子区推进综合配套和普兰店区域管理体制改革。

　　提升利用外资规模和水平。充分发挥先导区的拉动作用，创新招商机制，加大招商引资力度。全力抓好钻石海湾、普兰店湾、瓦房店沿海经济区、庄河临港经济区、旅顺绿色经济区、三十里堡等区域招商，形成新一轮项目引进高潮。加强对内招商，深化与国内大企业的战略合作，努力引进一批重点产业项目。

　　完善稳外需、保份额、促出口的各项政策措施。加强部门协作，提高通关效率。调整外贸结构，培育国家级产品出口基地，扩大自主知识产权、自主品牌和高技术含量、高附加值产品出口，促进外贸出口恢复性增长。

　　（五）着力统筹城乡发展，进一步壮大县域经济

　　加大统筹城乡发展力度，进一步夯实农业农村发展基础，使县域经济成为拉动经济增长的重要力量。

　　保持县域经济良好发展态势。加快发展县域工业，做强做大一批主业突出、关联性强的优势产业集群。着力抓好布局在县域的重大基础设施和产业项目建设，推动重点产业园区加速发展。加快县域商贸、旅游、物流和中介服务业发展，加大县域农贸市场、生产生活资料等专业市场及中心乡镇超市建设力度，健全市场体系。落实“三农”优惠政策，增加市级财政转移支付比重，支持县域经济快速发展。

　　发展都市型现代农业。坚持全域城市化与发展都市型现代农业并重，加快形成城乡统筹、工农相辅、集约发展的新格局。做强做优渔业、畜牧、水果、蔬菜和花卉业，推广国际标准，开拓国际市场。新发展设施农业11万亩、畜牧业标准饲养小区100处、标准化优质果园30万亩。抓好农产品质量安全和动物疫病防控，高标准创建一批都市型现代农业示范园区，建设大连国际花卉中心。加大农业科技创新和推广力度，推进农业机械化、信息化。

　　促进城镇化与新农村建设良性互动。完善县域城镇体系规划，依托现有城镇和产业园区，加强基础设施建设，提高教育、医疗、文化等基本公共服务能力，分层次、有重点地建设一批不同特色的城镇。推进户籍制度改革，增强城镇的集聚效应。推进新农村建设“六化”工程，抓好农村新社区和新村庄建设，加快农民住房改造，农村生态移民1000户。

　　（六）着力提升城市环境品质，加快建设“绿色大连”

　　围绕建设国际知名生态宜居城市的目标，坚持环境优先、生态立市，既要“金山银山”，更要绿水青山，努力走出一条生态文明与经济建设高度融合的绿色发展之路。

　　完善城市基础设施。加快推进大伙房水库输水入连、体育中心、铁路大连北站、京大客运专线、丹大铁路、202路轨道延伸线、庄盖路、振连路等工程建设，启动长海机场扩建和跨海大桥、金州至普兰店快轨工程。推进红沿河核电二期前期工作。拓宽改造联合路、胜利路东段和解放路南段，开工建设西部通道南段立交桥等工程，下大气力促进道路畅通。

　　强化生态建设和环境保护。加快创建国家森林城市，完成滨海大道二期等十大重点绿化美化工程，推进西城、大连湾、大黑山森林公园和英歌石植物园、西山公园建设，实施老虎滩、傅家庄等滨海公园升级改造。城乡植树1亿株，城市绿化覆盖率达到45%，人均公共绿地达到13平方米。加大河道生态工程建设力度。加强海域使用管理，搞好海洋环境保护。加快建立城乡新型垃圾除运体系和生活污水处理体系。

　　加大节能减排力度。制定产业能效指南，严格控制高耗能项目建设。大力发展新能源和可再生能源，加快建设以低碳排放为特征的产业体系和消费模式。继续抓好节能与新能源汽车推广示范工程，搞好建筑节能。发展循环经济，培育静脉产业。加强对重点减排项目的督办与监管。启动14座城镇污水处理厂提标工作，进一步提高中水回用率，完成废水不达标重点企业的治理整改，统一治理电镀企业污染。开展大气环境综合整治，继续实施拆炉并网工程。

　　提升城市管理水平。按照“全新标准、全域覆盖、全面考核”要求，加强城市管理。开展市容环境综合整治，改变建设工地混乱状况，修缮建筑立面、市政设施和公共设施。采取切实有效措施，基本解决车辆乱停乱放问题。实施居民小区规范化管理，维修治理背街小巷，为广大市民创造井然有序、干净整洁的居住环境。

　　（七）着力发展各项社会事业，促进社会和谐稳定

　　着眼确保公益、促进均衡，提高社会事业发展水平，努力满足人民群众基本公共需求，全面建设和谐大连。

　　促进教育均衡发展。做好教育资源布局规划与调整，推进住宅小区配套学校建设，加强薄弱学校改造，扩建市第十一中学、第二中学，新建大连市高中综合实践活动基地，筹建大连市职业教育基地，对农村困难家庭学生实施免费职业教育。加强师德建设，提高教师专业水平。发展民办教育、学前教育、特殊教育、远程教育。支持在连高校加快发展。

　　全面落实医改任务。健全基层医疗卫生服务体系，在公办社区卫生服务机构和乡镇卫生院实行国家基本药物制度。新建儿童医院和妇产医院，改造市结核病医院。新农合最低年筹资标准提高到人均160元以上，最高支付限额提高到农民人均纯收入的6倍。提高疾病预防控制能力和卫生信息化应用水平。科学规划人口布局，促进人口均衡发展，搞好第六次全国人口普查。

　　增强城市文化软实力。加快国际会议中心、人民剧场、现代博物馆、汉墓博物馆、贝壳博物馆等公共文化设施的建设和改造。全面完成农家书屋和农村文化信息资源共享工程，推进数字图书馆“一卡通”工程。积极稳妥推进文化体制改革，加快文化产业发展，加大文化遗产保护力度。认真贯彻全民健身条例。提高竞技体育水平，重塑大连足球等优势项目的强者形象，做好参加省运会和备战全国城运会各项工作。

　　加强社会管理创新。实施重点行业和领域安全生产专项整治，开展“中国安全保障型城市示范区”创建工作，坚决防范遏制重特大事故发生。加强防震减灾和防汛抗旱工作，提高全市综合减灾能力。强化食品药品安全监管，建设食品安全城市。推进基层民主政治建设，完成第十届农村村委会换届选举。

　　切实维护社会稳定。建立和完善“大信访”、“大调解”体制机制，实施联合接访，化解矛盾纠纷。加强应急预案体系、应急队伍和应急平台建设，提高应急管理能力。加强法制宣传和法律援助工作，全面落实“五五”普法任务。严厉打击违法犯罪活动，推进治安防范综合治理社会化。

　　继续做好民族、宗教、外事、侨务、对台、人防、气象、档案、新闻出版、双拥共建以及民兵和预备役等工作。

　　（八）着力保障和改善民生，提高人民群众生活水平

　　扎实推进重点民生工程，全市筹集资金50亿元，为人民群众办好15件实事，切实解决人民群众最关心、最直接、最现实的利益问题。

　　打造充分就业城市。出台创业指导实施意见，加快创建国家级创业型城市，建立中国（大连）创业者实训基地，以创业带动就业。实现实名制就业16万人，确保零就业家庭动态为零。深入实施特别培训计划，培训各类人员7万人。加强人才储备，保持各级政府储备人才2000人以上。

　　提高社会保障水平。扩大社会保障覆盖范围，出台城镇无社会保障老年居民养老保障暂行办法和养老保险关系转移接续实施办法。实行城镇居民医疗保险市级统筹。提高最低工资标准、失业保险和企业退休人员养老金待遇水平。推进残疾人社会保障和服务体系建设。

　　健全城乡社会救助体系。落实城乡低保标准自然增长机制，出台城乡居民普惠性临时救助政策，实现社会救助全覆盖。启动城乡社区养老服务中心建设三年规划，推进社会福利事业加快发展。建立亿元慈善基金长效救援机制，扩大慈善救助规模。扎实开展农村定点帮扶工作。

　　加快住房保障体系建设。加强中小套型、中低价位商品住房和创业租赁房建设，通过货币补贴方式安排经济适用房8000户，对符合廉租住房条件的家庭实现应保尽保。稳步提高住房公积金缴存基数和比例，进一步扩大覆盖面和惠及面。加强监管调控，规范交易、租赁、置换等行为，保持房地产市场平稳发展。

　　强化公共服务。继续抓好城建“五个一”工程。加快三道沟净水厂改扩建，一次性完成185公里城市供水旧管网改造，新建农村安全自来水工程95项、解决10万人饮水安全。加大煤气管网建设力度，分两年完成300公里旧煤气管网改造工程，解决煤气配套历史遗留问题。开辟、延伸、延时8条公交线路。继续做好农村客运线路集约化改造。深入实施早餐工程和菜篮子便利工程。完善城乡蔬菜、粮食市场体系，努力稳定农副产品价格。

　　（九）着力提高行政效能，建设人民满意政府

　　面对复杂形势和繁重任务，我们必须增强领导科学发展的能力，切实把服务型政府建设的各项工作落到实处。

　　始终保持发展激情。各级政府和广大公务员必须胸怀大局、目标高远，切实增强勇立潮头、跨越发展的信心，真正肩负起新的历史使命和责任；必须抢抓机遇、乘势而上，采取超常举措，务求干则必成，努力抢占新一轮发展的制高点；必须锐意创新、开拓进取，用世界眼光和战略思维科学谋划大连未来发展，不断开创振兴发展的新局面。

　　全面提高行政效能。进一步减少和规范行政审批事项，加强和改进“一站式”服务，市级审批项目网上审批达到100%。深入推进政府信息公开，加强电子政务建设，提高政府工作透明度、公信力和服务效率。强化政务督查、效能监察和绩效考评，确保政令畅通和工作落实。自觉接受人大法律监督、政协民主监督、群众和舆论监督，做好人大代表议案、建议和政协提案办理工作。

　　切实转变工作作风。树立科学发展的政绩观，决不搞脱离实际、劳民伤财的“形象工程”和“政绩工程”。大兴调查研究、求真务实、埋头苦干之风，坚持深入基层、深入实际，努力发现和解决新问题。发扬曹连新精神，锲而不舍，艰苦奋斗，攻坚克难，争创一流工作业绩。

　　深入抓好廉政建设。坚持依法行政、从严治政，强化审计监督、行政监察，确保行政权力依法、公正、有效运行。严格规范建设工程招投标、政府采购、国有资产转让等公共资源管理，严肃查处违法违纪案件，坚决纠正不正之风，努力消除腐败现象，营造风清气正、廉洁高效的政务环境。

　　各位代表！大连已经进入了科学发展、创新发展、和谐发展的新时代。让我们紧密团结在以胡锦涛同志为总书记的党中央周围，在中共大连市委的领导下，满怀发展激情，焕发昂扬斗志，恪尽职守，不辱使命，为加快率先全面振兴、实现科学发展新跨越而努力奋斗！
